# The NlpD Lipoprotein Is a Novel *Yersinia pestis* Virulence Factor Essential for the Development of Plague

**DOI:** 10.1371/journal.pone.0007023

**Published:** 2009-09-14

**Authors:** Avital Tidhar, Yehuda Flashner, Sara Cohen, Yinon Levi, Ayelet Zauberman, David Gur, Moshe Aftalion, Eytan Elhanany, Anat Zvi, Avigdor Shafferman, Emanuelle Mamroud

**Affiliations:** Department of Biochemistry and Molecular Genetics, Israel Institute for Biological Research, Ness-Ziona, Israel; Institut Pasteur, France

## Abstract

*Yersinia pestis* is the causative agent of plague. Previously we have isolated an attenuated *Y. pestis* transposon insertion mutant in which the *pcm* gene was disrupted. In the present study, we investigated the expression and the role of *pcm* locus genes in *Y. pestis* pathogenesis using a set of isogenic *surE, pcm, nlpD* and *rpoS* mutants of the fully virulent Kimberley53 strain. We show that in *Y. pestis, nlpD* expression is controlled from elements residing within the upstream genes *surE* and *pcm*. The NlpD lipoprotein is the only factor encoded from the *pcm* locus that is essential for *Y. pestis* virulence. A chromosomal deletion of the *nlpD* gene sequence resulted in a drastic reduction in virulence to an LD_50_ of at least 10^7^ cfu for subcutaneous and airway routes of infection. The mutant was unable to colonize mouse organs following infection. The filamented morphology of the *nlpD* mutant indicates that NlpD is involved in cell separation; however, deletion of *nlpD* did not affect *in vitro* growth rate. Trans-complementation experiments with the *Y. pestis nlpD* gene restored virulence and all other phenotypic defects. Finally, we demonstrated that subcutaneous administration of the *nlpD* mutant could protect animals against bubonic and primary pneumonic plague. Taken together, these results demonstrate that *Y. pestis* NlpD is a novel virulence factor essential for the development of bubonic and pneumonic plague. Further, the *nlpD* mutant is superior to the EV76 prototype live vaccine strain in immunogenicity and in conferring effective protective immunity. Thus it could serve as a basis for a very potent live vaccine against bubonic and pneumonic plague.

## Introduction

*Yersinia pestis* is the etiological agent of plague, which has caused millions of deaths in three world pandemics and is still a public health issue in some regions of the world. The most prevalent form of the disease is the bubonic plague, which develops following transmission of the pathogen from rodent reservoirs to humans via infected fleas [1]. Primary pneumonic plague is less abundant in nature and results from inhalation of *Y. pestis* droplets or aerosols. It is a rapidly progressing disease leading to high mortality rates in untreated patients and can spread from person to person [1]. These characteristics led to the recognition of *Y. pestis* as a potential threat agent [2].

The ability of *Y. pestis* to respond to the host environment and to overcome immune systems is attributed to the combined activity of multiple virulence mechanisms. Among these mechanisms, only few have been found to be absolutely required for virulence in animal model systems. The type III secretion system (TTSS) is essential for survival of the pathogen within the mammalian host environment. This was demonstrated by the inability of *Y. pestis* strains devoid of the plasmid carrying the TTSS (pCD1^−^) genes to colonize host tissues and to produce systemic disease following infection via both subcutaneous (s.c.) and airway routes [3]–[6]. The TTSS, shared by the closely related enteropathogens *Y. enterocolitica* and *Y. pseudotuberculosis*, comprises a secretion apparatus, chaperones and several effectors (Yops) and leads to the modulation of cell signaling networks necessary for an effective immune response [7], [8]. Other virulence factors were found to be indispensable for *Y. pestis* pathogenesis via the s.c. route of infection. These include the plasminogen activator factor, which is encoded on the pPCP1 plasmid [9]–[11]; the Yersiniabactin (Ybt) iron acquisition system, which is encoded within the high pathogenicity island [12], [13]; the chromosomally encoded PurH involved in the synthesis of purines [4], [14]; adenylate kinase, which is involved in nucleotide metabolism [15]; and the recently characterized YadBC [16].

**Figure 1:**
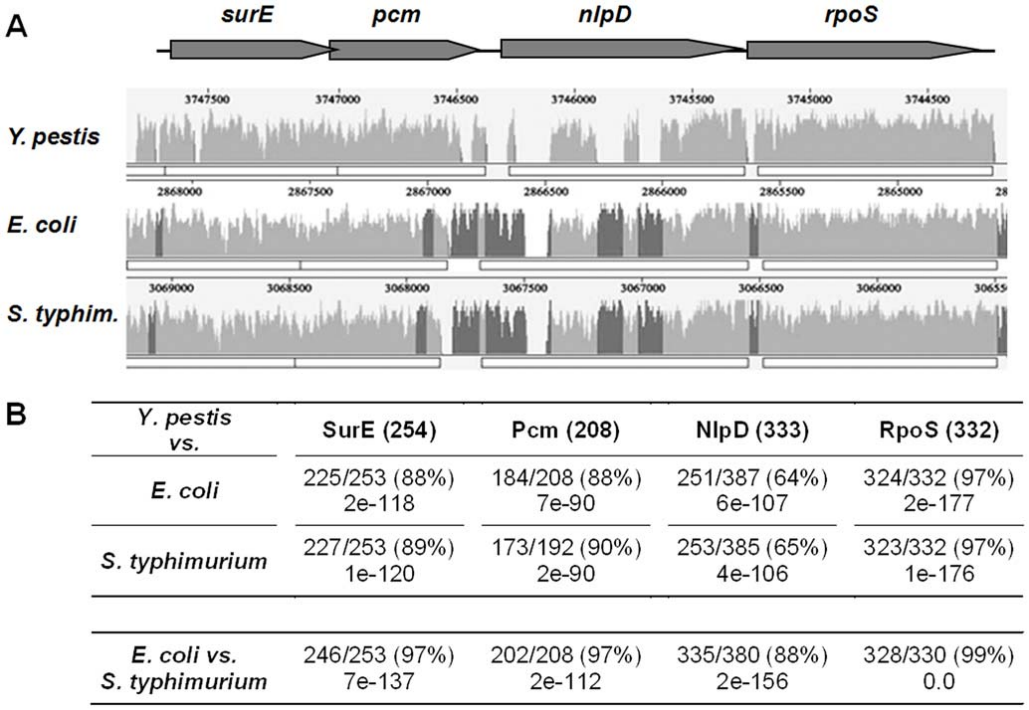
*In silico* analysis of the *pcm* genomic locus of *Y. pestis*. (A) A similarity plot representing the alignment of the *Y. pestis* CO92 *surE*, *pcm*, *nlpD* and *rpoS* genomic locus (gray arrows) with the corresponding orthologous regions of *E. coli* K12 and *S. typhimurium* LT2. Peaks represent regions of sequence conservation; regions that are conserved among all three genomes are in light gray, while regions that are conserved only between *E. coli* and *S. typhimurium* are in dark gray. (B) Similarity comparison of the amino acid sequences of the *Y. pestis* CO92 *pcm* locus-encoded proteins to the corresponding proteins in *E. coli* and *S. typhimurium. Y. pestis* protein length is indicated in parenthesis and the similarity and E-values with respect to the *E. coli* or *S. typhimurium* proteins are provided. In the bottom row, the similarity between the *E. coli* and *S. typhimurium* proteins is presented.

In a previous study, we isolated a highly attenuated mutant designated Kimberley53*pcm* (Kim53-K9) in which the *pcm* gene was disrupted by a mini-Tn5 transposon insertion [4]. When administered subcutaneously to mice, colonization and persistence of Kim53-K9 in the spleen and liver were substantially reduced and the median lethal dose (LD_50_) of the mutant was more than seven orders of magnitude higher than the LD_50_ of the wild type Kimberley53 strain [4]. The homologous *E. coli pcm* gene codes for a Protein-L-isoaspartate O-methyltransferase (Pcm), which can specifically catalyze the transfer of the methyl groups from S-adenosylmethionine to atypical L-isoaspartyl residues in proteins [17], [18]. The protein was found to be involved in the survival of stationary phase cells when exposed to environmental stress conditions [18]. Immediately upstream of the *E. coli pcm* gene lies the *surE* gene (Fig. 1), which overlaps with *pcm* by four nucleotides. In *E. coli*, these two genes share a bicistronic operon, but *pcm* can be transcribed independently from its own promoter [19]. The *nlpD* gene is located downstream of *pcm* and codes for an outer membrane lipoprotein that is assumed to be involved in cell wall formation and maintenance [20], [21]. The last gene in the locus is *rpoS*, which encodes an alternative RNA polymerase Sigma factor (RpoS). The *rpoS* and the *nlpD* genes constitute an operon; however, the major *rpoS* promoter is located within the *nlpD* gene [22]–[25]. The expression of *rpoS* is induced during stress conditions, such as starvation and extreme pH, and during stationary growth phase [26], [27]. This factor has been found to be involved in *S. typhimurium* virulence in a mouse infection model [28].

Genes within the *pcm* “stress locus” were extensively studied in many species of the *Enterobacteriaceae* family, including *Yersinia enterocolitica* [29], [30]. However, their importance for the pathogenesis of plague has not been evaluated so far. In the present study, we have characterized the *pcm* genomic locus in *Y. pestis*. The expression pattern of the genes was evaluated along with their respective contributions to *Y. pestis* virulence using mouse models of bubonic and pneumonic plague. Our findings indicate that within the *pcm* locus, *nlpD* is the only essential gene for *Y. pestis* virulence. In addition, a highly attenuated *Y. pestis nlpD*-null mutant has been shown to induce an efficient protective immunity against bubonic and pneumonic plague.

## Results

### The *pcm* locus of *Y. pestis*

In a previous screen designed to isolate attenuated *Y. pestis* Kimberley53 mutants, a transposon insertion was identified at the 3′ end of the *pcm* gene of the Kim53-K9 mutant [4]. When administered subcutaneously to mice, the virulence of Kim53-K9 was severely attenuated [4]. Inspection of the genome sequence of *Y. pestis* CO92 [31] revealed that the *pcm* locus shows high conservation of the gene order with respect to the related enteropathogens *E. coli* and *Salmonella* (Fig. 1A). However, whereas the *Y. pestis* SurE, Pcm and RpoS proteins share high levels of identity with their related *E. coli/Salmonella* proteins (88–97%), the NlpD lipoproteins are more divergent (64–65%, Fig. 1B). The most noticeable disparity between the NlpDs of *Y. pestis* and *E. coli/Salmonella* is a protein sequence containing a unique proline and glutamine-rich repeat region at the N-terminus that is present in the proteins of the enteric pathogens but is completely absent from NlpD of *Y. pestis* and all other *Yersinia* species. Examination of the NlpD sequences within the *Yersinia* genus revealed that the *nlpD* gene products in *Y. pseudotuberculosis* and *Y. enterocolitica* have relatively high levels of sequence similarity to the corresponding gene product of *Y. pestis* (98% and 94%, respectively).

**Figure 2:**
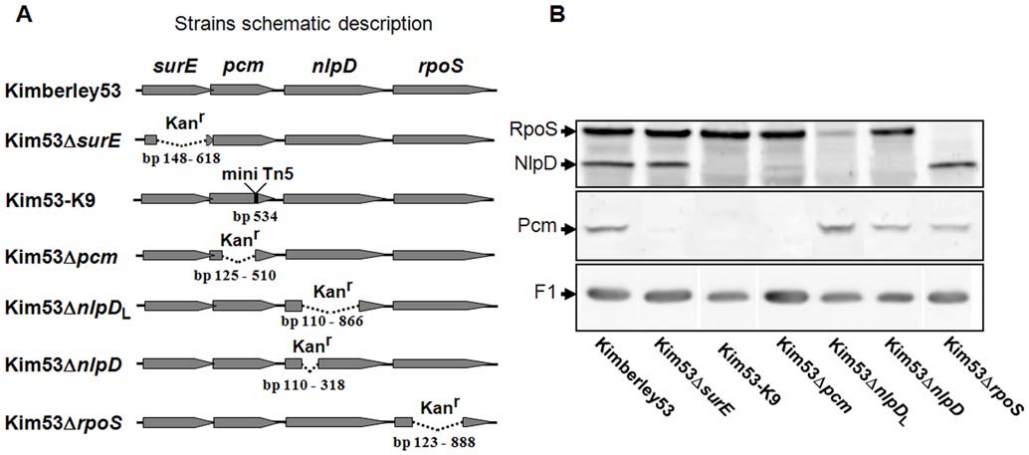
Expression of *pcm*, *nlpD* and *rpoS* in *Y. pestis* derivatives. (A) Schematic description of *Y. pestis* mutants. A kanamycin resistance cassette (Kan^r^) was inserted in place of the deleted region. In all mutants the Kan^r^ cassette is oriented in the same direction as the *pcm* locus genes and the runthrough transcription is minimal as evident from the expression analyses of Kim53Δ*surE*, Kim53Δ*pcm* and Kim53Δ*nlpD*_L_. (B) Assessment of bacterial Pcm, NlpD and RpoS expression. Cultures of the *Y. pestis* strains were inoculated (initial OD_660_ = 0.01) and incubated for an additional 24 hours at 37°C. Western blot analysis was performed with anti-Pcm, anti-NlpD and anti-RpoS antibodies. F1 expression levels in the indicated strains were detected with anti-F1 antibodies and are shown to indicate that comparable amounts of bacterial extracts were blotted on the membrane.

To characterize the factor/s involved in the attenuated phenotype of Kim53-K9, we used the virulent *Y. pestis* Kimberley53 strain to generate a series of isogenic deletion mutants within the *pcm* locus genes (Table 1). First, the DNA sequence of the *pcm* locus of Kimberley53 was determined (GenBank acc. no. FJ666123) and was found to be identical to CO92. In each of the newly constructed mutants, a defined region of a single gene was deleted and replaced with a kanamycin resistance cassette (see details in “Materials and Methods”), resulting in *Y. pestis* Kimberley53 derivatives that were designated Kim53Δ*surE*, Kim53Δ*pcm*, Kim53Δ*nlpD* and Kim53Δ*rpoS* (Fig. 2A). PCR analysis verified that all of the Kimberley53-derived strains carry the pMT1, pCD1, pPCP1 plasmids and the chromosomal *pgm* locus. To evaluate the expression of Pcm, NlpD and RpoS in these strains, bacterial cultures were grown to stationary phase (24 hours) at 37°C and 28°C in heart infusion broth (HIB) and then subjected to Western blot analysis with highly specific antibodies. The patterns of expression were independent of the growth temperature (data not shown). In the Kim53Δ*surE* mutant, Pcm was not detected, whereas the levels of NlpD and RpoS were comparable to the wild type strain (Fig. 2B). This result suggests that a control element influencing *pcm* expression resides within the *surE* gene, as has been reported for *E. coli pcm* [19]. In the Kim53-K9 strain, Pcm and NlpD were not detected, whereas the level of RpoS was comparable to the wild type strain (Fig. 2B). This finding suggests that expression of *nlpD* is regulated by an element within the *pcm* gene. This assumption was further supported by the observation that the level of NlpD was reduced in Kim53Δ*pcm*, whereas that of RpoS was not altered. Of note, in *E. coli* and *Salmonella*, expression of *nlpD* is driven by promoters located within the intergenic region between *pcm* and *nlpD* [21], [22], [25], [27], [32]. The genomic sequence in this region differs considerably between *Y. pestis* and the enteric pathogens (Fig. 1A). The *rpoS* gene resides downstream of *nlpD*. In bacterial enteropathogens, the major *rpoS* promoter has been identified within the *nlpD* gene [33 and references therein], [34], [35]. We attempted to locate these known enterobacterial *rpoS* promoter sequences in the corresponding *Y. pestis* Kimberley53 and CO92 genomic regions. Putative *rpoS* promoter elements within the *nlpD* gene (547 bp upstream of the ATG start codon of *rpoS*) were identified according to tandem presence of −35 and −10 consensus sequences (Fig. S1). We therefore constructed two *nlpD*-null mutants: KimΔ*nlpD*_L,_ in which the putative *rpoS* promoter elements were deleted, and KimΔ*nlpD*, in which these sequences were preserved (Fig. 2A). As expected, the level of RpoS was decreased in KimΔ*nlpD*_L_ but not in KimΔ*nlpD* (Fig. 2B). Of note is that the Kan^r^ phenotype does not appear to derive expression of downstream ORFs, see for example the expression levels of Pcm in Kim53Δ*surE*, NlpD in Kim53Δ*pcm*, and RpoS in Kim53Δ*nlpD*_L_.

**Table 1:** *Y. pestis* strains and plasmids used in this study.

Strain or plasmid	Relevant characteristic(s)	Reference or source
*Y. pestis* strains		
Kimberley53	Virulent strain	[57]
EV76	*pgm*- (Girard's strain)	[58]
Kim53-K9	Kimberley53 strain in which the mini-Tn5 transposon was inserted into the *pcm* gene (nt no. 532 in the coding sequence).	[4]
* *Kim53Δ*surE*	Kimberley53 strain in which bp 148 to 618 (out of 765) of the *surE* gene were deleted; *kan* ^R^	This study
Kim53Δ*pcm*	Kimberley53 strain in which bp 125 to 510 (out of 624) of the *pcm* gene were deleted; *kan* ^R^	This study
* *Kim53Δ*nlpD_L_*	Kimberley53 strain in which bp 112 to 866 (out of 999) of the *nlpD* gene deleted; *kan* ^R^	This study
* *Kim53Δ*nlpD*	Kimberley53 strain in which bp 112 to 318 (out of 999) of the *nlpD* gene were deleted; *kan* ^R^	This study
* *Kim53Δ*rpoS*	Kimberley53 strain in which bp 123 to 888 (out of 996) of the *rpoS* gene were deleted; *kan* ^R^	This study
Kim53Δ*yopJ*	Kimberley53 deleted in *yopJ*	[59]
Plasmid		
p*nlpD*	The complete *nlpD* coding sequence and the 442 bp region directly upstream were cloned into the pBR322 plasmid (Promega); *amp* ^R^	This study

The last gene in the *pcm* locus that was analyzed was *rpoS*, which is predicted to encode an alternative sigma factor expressed during stress conditions [36]. The Pcm and NlpD expression levels in Kim53Δ*rpoS* were comparable to the levels found in the wild type strain (Fig. 2B). Likewise, in *E. coli*, expression of the *pcm* and *nlpD* genes is RpoS-independent [19], [37].

### Identification of the sites of transcription initiation of the *pcm, nlpD* and *rpoS* genes of *Y. pestis* and their relation to the observed expression profiles

**Figure 3:**
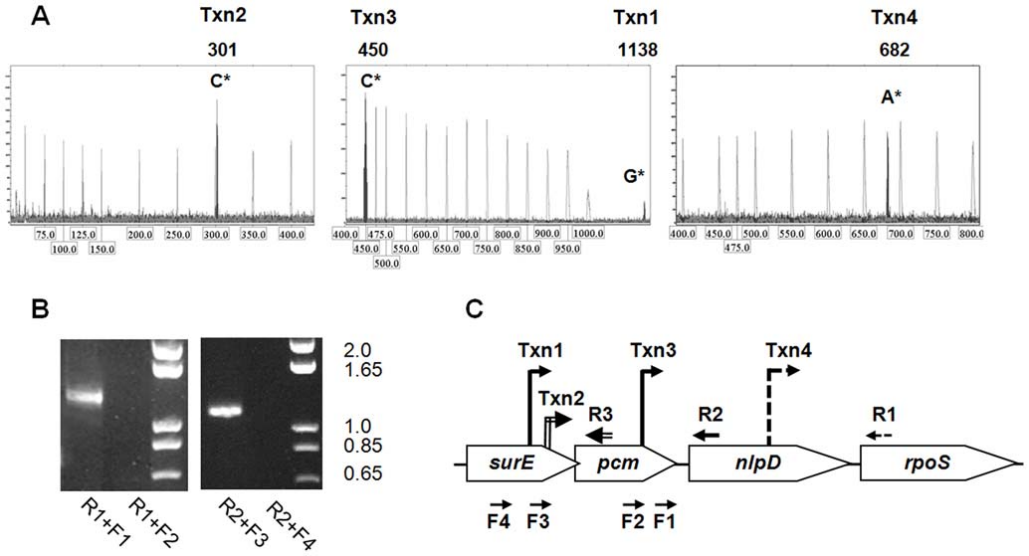
Identification of *Y. pestis pcm, nlpD* and *rpoS* transcription start sites. (A) Primer extension (PE) electropherogram used to analyze the PE product generated using primers R1 [*rpoS*-rev (137)], R2 [*nlpD*-rev (151)] or R3 [*pcm-*rev (126)] (Table S1). PE peaks (black) corresponding to the MapMarker®1000 internal size standards [gray, see actual size (bp) at the bottom of the electropherogram] are marked with the related nucleotide from the Kimberley53 genomic sequence. The size of the PE product is indicated at the top of the electropherogram. (B) RT-PCR products generated with the reverse primers specified above and the forward primers: F1 [*rpoS*-for (−1355)], F2 [*rpoS*-for (−1493)], F3 [*nlpD*-for (−972)] and F4 [*nlpD*-for (−1389]. (C) Positions of R1-R3 primers used for RT-PCR and PE analyses is indicated by dashed, solid and double lines, respectively. The position of transcription start sites determined according to the PE products length (Txn 1–4), and the position of the F1-F4 primers used for RT-PCR are indicated.

To further characterize the elements regulating the expression of the *pcm, nlpD* and *rpoS* genes, total RNA was prepared from cultures of *Y. pestis* bacteria grown to stationary phase (24 hours) at 37°C and then was used for primer extension analysis and RT-PCR. Using a primer complementary to the 5′ region of the *pcm* gene, a single transcription initiation site designated Txn2 was identified within the *surE* gene, 176 bp upstream of the ATG start codon of the *pcm* gene (Fig. 3A left panel). Similar analysis performed with a primer complementary to the 5′ region of *nlpD* allowed identification of two transcriptional initiation sites within the *surE* and *pcm* genes located 988 bp (Txn1) and 300 bp (Txn3) upstream of the ATG start codon of *nlpD*, respectively (Fig. 3A middle panel). To corroborate these findings, RT-PCR analysis was performed using primers complementary to regions within the *nlpD* and *surE* genes. An 1123-bp fragment was observed extending from the *nlpD* gene to the *surE* gene at a site between Txn1 and Txn2 (Fig. 3B right panel, R2+F3). This result indicates that a bicistronic *pcm*-*nlpD* message is indeed transcribed from Txn1. No message could be detected using primers located upstream of Txn1 (Fig. 3B right panel, R2+F4). RT-PCR analysis performed using primers complementary to regions within the *rpoS* and *pcm* genes indicated that Txn3 drives transcription of an *nlpD-rpoS* bicistronic message (Fig. 3B left panel, R1+F1). No RNA message could be detected using primers located upstream of Txn3 (Fig. 3B left panel, R1+F2). The major *rpoS* transcription initiation start site (Txn4) was identified within the *nlpD* gene at the exact location predicted by the *in silico*-analysis (Fig. S1 and Fig. 3A right panel).

Integration of the data obtained from primer extension, RT-PCR and Western blot analyses (Fig. 2 and Fig. 3) helped to unravel the complex regulation controlling the expression of *Y. pestis pcm, nlpD* and *rpoS* genes (Fig. S2). Transcription of the *pcm* gene is driven by control elements within the *surE* gene, and a major transcription start site was identified at Txn2 (Fig. 3C). Consistent with this result, Pcm was not detected in the Kim53Δ*surE* mutant (see Fig. 2). Transcription of the *nlpD* gene can start in principle from three different positions: Txn1, Txn2 and Txn3. However, in our analysis we failed to see *nlpD* transcripts from Txn2. Transcription starting at position Txn1 leads to the synthesis of a *pcm-nlpD* bicistronic message, whereas transcription starting from position Txn3 drives the synthesis of an *nlpD-rpoS* bicistronic message (Fig. 3C). The finding that the NlpD level was severely reduced in the Kim53Δ*pcm* mutant in which the genomic region containing Txn3 was deleted (Fig. 2) indicates that this region includes a major *nlpD* control element/s. In agreement with this result, deletion of the Txn1 genomic region hardly interfered with NlpD expression (see Fig. 2, Kim53Δ*surE*). Putative *Y. pestis rpoS* promoter sequences were identified within the *nlpD* gene by *in-silico* analysis (Fig. S1), and the *rpoS* transcription start point was indeed identified at that region (Txn4, Fig. 3C). A significant reduction in the RpoS level was observed upon deletion of the Txn4 region, indicating that this region indeed contains major *rpoS* control elements (see Fig. 2, Kim53Δ*nlpD*_L_). The low RpoS level detected in Kim53Δ*nlpD*_L_ (Fig. 2) probably represents the basal expression level of the *nlpD-rpoS* bicistronic message transcribed from Txn3 (see Fig. 3C). In accordance with these results, a deletion generated within the *nlpD* gene that did not affect Txn4 did not cause a significant reduction in the RpoS level (see Fig. 2 Kim53Δ*nlpD*).

**Figure 4:**
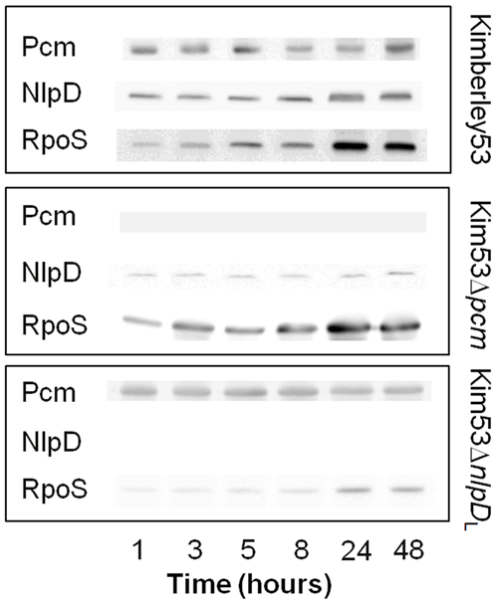
Growth phase-dependent expression of Pcm, NlpD and RpoS in *Y. pestis*. Western blot analysis of bacterial extracts prepared from culture samples taken at the indicated time points was performed with anti-NlpD, anti-RpoS and anti-Pcm antibodies.

To characterize the expression patterns of the *Y. pestis pcm, nlpD* and *rpoS* genes during logarithmic and stationary growth phases, wild type Kimberley53 and the Kim53Δ*pcm* and Kim53Δ*nlpD* mutants were grown in HIB for 48 hours, and equal amounts of total protein were loaded on sodium dodecyl sulfate-polyacrylamide gel electrophoresis (SDS-PAGE). Western blot analysis of Kimberley53 cells indicated that Pcm and NlpD levels remained essentially constant during the transition from logarithmic to stationary growth phase, whereas the RpoS expression pattern was growth phase-dependent (Fig. 4). One should note that during the stationary growth phase an NlpD precursor starts to accumulate, appearing as a slightly slower migrating band than NlpD. Similar findings were reported for *E. coli* [21], [36], [38]. The Western blot analyses with the various mutants (Fig. 4), together with the RT-PCR results (Fig. 3B), indicate that the low expression levels of NlpD and RpoS were governed by distal control elements (Txn1 and Txn3, respectively). We therefore evaluated the contribution of each of the distal elements to NlpD (Txn1) and RpoS (Txn3) expression *in vitro* during growth. In Kim53Δ*pcm*, the NlpD level was constant but was lower than that of the wild type strain (Fig. 4). Consequently, the region in proximity to Txn3 contains the major *nlpD* control element. In Kim53Δ*nlpD*_L_, in which the Txn4 region was deleted, the level of RpoS was low and growth phase-dependent (Fig.4). This finding indicates that the major control elements regulating RpoS expression are located in close proximity to the Txn4 genomic region.

### NlpD is an essential factor for the development of bubonic and pneumonic plague

**Figure 5:**
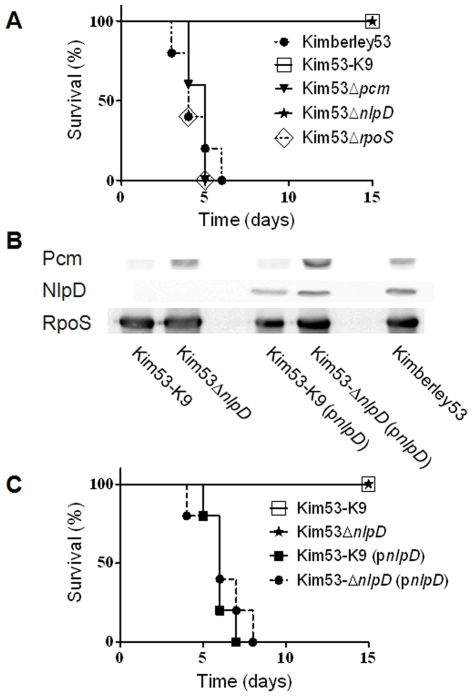
Virulence of *Y. pestis pcm* locus mutants in mice. (A) Virulence of the *Y. pestis pcm* locus mutants in the mouse model of bubonic plague. Groups of five mice were infected subcutaneously with 100 cfu/mouse of the indicated *Y. pestis* strains; Kimberley53 (closed circles), Kim53-K9 (open squares), Kim53Δ*pcm* (closed triangles), Kim53Δ*nlpD* (stars) and Kim53Δ*rpoS* (open diamonds). (B) Episomal expression of *nlpD* in attenuated *Y. pestis* mutants. Western blot analysis of the indicated cultures was performed as described in the legend to Figure 2B (C) Virulence of the *nlpD*-complemented *Y. pestis* mutants in the mouse model of bubonic plague. Groups of five mice were infected subcutaneously with 100 cfu/mouse of the indicated *Y. pestis* strains; Kim53Δ*nlpD* (stars), Kim53-K9 (open squares), Kim53Δ*nlpD*(p*nlpD*) (closed squares) and Kim53-K9(p*nlpD*) (closed circles).

To test the contribution of SurE, Pcm, NlpD and RpoS to *Y. pestis* pathogenesis, we evaluated the virulence of Kimberley53-deletion mutants in mouse models of bubonic plague (s.c. infection) and pneumonic plague (intranasal infection). In the bubonic plague mouse model, Kim53Δ*surE*, Kim53Δ*pcm* and Kim53Δ*rpoS* were found to be highly virulent as reflected by the mortality rate, the mean time to death and the LD_50_ value (Fig. 5A, Table 2). In contrast, The Kim53-K9 and Kim53Δ*nlpD* strains were avirulent (Fig. 5A, Table 2).

**Table 2:** Virulence of *Y. pestis* strains in mouse models of bubonic and pneumonic plague.

*Y. pestis* strain	LD_50_ valuea,b (cfu)	
	s.c. route	i.n. route
Kimberley53	1–3	550
Kim53-K9	>1×10^7^	ND
Kim53Δ*surE*	<1×10^2^	ND
Kim53Δ*pcm*	3	3300
Kim53Δ*nlpD*	>2×10^7^	>4×10^7^
Kim53Δ*nlpD*(p*nlpD*)	<1×10^2^	<1500
Kim53-K9(p*nlpD*)	<1×10^2^	ND
Kim53Δ*rpoS*	3	1100

ND = not determined.

a The “<” symbol indicates that the calculated LD_50_ value is the minimal infection dose tested, under which more than 50 percent of the animals died.

b The “>” symbol indicates that the calculated LD_50_ value is the maximal infection dose tested under which less than 50 percent of the animals died.

The inability of Kim53-K9 and Kim53Δ*nlpD* to overcome host defense systems was further emphasized by the finding that infecting mice with higher doses of these mutants, up to 10^7^ cfu of each strain, was non-lethal (Table 2) and that infected mice did not show any visible disease symptoms, such as ruffled hair or a hunched back.

Intranasal (i.n.) infection of mice was demonstrated recently to serve as a model for studying the development of primary pneumonic plague [3]. We therefore used this model system to study the involvement of our genes of interest in development of pneumonic plague. All mice infected intranasally with 2×10^4^ cfu of wild type Kimberley53 succumbed to the infection by day 4 (data not shown), and the LD_50_ was found to be 550 cfu (Table 2). Similar LD_50_ values were reported recently for other virulent *Y. pestis* strains [39], [40]. The Kim53Δ*pcm* and Kim53Δ*rpoS* mutants were able to produce disease upon i.n. infection of mice, and the LD_50_ values of these mutants were 6 and 2 fold higher (respectively), than the LD_50_ of the wild type strain (Table 2). In contrast, i.n. infection of mice with up to 4×10^7^ cfu of Kim53Δ*nlpD* was non-lethal (Table 2), and infected mice did not show disease symptoms.

To substantiate our observation that the attenuated phenotype of the NlpD-null mutants (Kim53Δ*nlpD* and Kim53-K9) resulted from the loss of NlpD expression, we examined the ability of a trans-complemented *Y. pestis nlpD* gene to restore the mutant's virulence. Due to the toxicity associated with over-expression of NlpD ([21] and our unpublished data), we expressed the gene using its native control element. The complete coding sequence of the *Y. pestis* Kimberley53 *nlpD* gene preceded by the 442 bp upstream of the *nlpD* ATG, a region that includes the Txn3 transcription initiation region, was cloned into pBR322. The plasmid was introduced into Kim53Δ*nlpD* and Kim53-K9 to give the complemented strains Kim53Δ*nlpD*(p*nlpD*) and Kim53-Κ9(p*nlpD*). Western blot analysis performed using an overnight culture of the complemented strains grown at 37°C or 28°C (not shown) confirmed that in both strains the NlpD expression level was comparable to expression in the wild type strain (Fig. 5B). All mice infected subcutaneously with 100 cfu of the NlpD-complemented strains (the bubonic infection model) succumbed to the infection by day 8 (Fig. 5C). Moreover, Kim53Δ*nlpD*(p*nlpD*) also regained virulence following i.n. infection (the pneumonic infection model) (Table 2). These results confirmed that NlpD is a novel virulence factor of *Y. pestis* and is essential for development of both bubonic and pneumonic plague. Finally, NlpD expression on the background of the Kim53-K9 mutant also restored virulence, confirming that the attenuation of Kim53-K9 was not due to the absence of the *pcm* gene product but resulted from the elimination of *nlpD* expression.

### Deletion of *nlpD* impairs *Y. pestis* colonization of internal organs in mice

**Figure 6:**
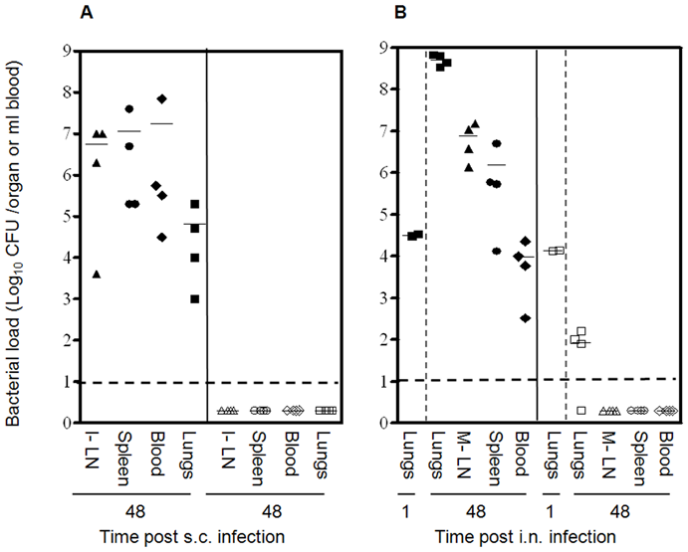
Colonization of mouse organs by *Y. pestis* strains. (A) Bacterial colonization of mouse organs after s.c. infection. Mice were injected subcutaneously with either 1×10^4^ cfu of Kimberley53 (closed symbols) or 1×10^5^ cfu of Kim53Δ*nlpD* (open symbols). Animals were sacrificed at 48 h post infection. Blood was collected and the draining inguinal lymph nodes (I-LN), the spleen and the lungs were harvested from each mouse, homogenized in 1 ml PBS and cultured on BHIA plates at 28°C for 48 hours. Values represent total bacteria in the organs (cfu/organ), or the bacterial concentration in blood (cfu/ml). The dashed line indicates the limit of detection. Horizontal bars represent the average value of the bacterial load in each case. (B) Bacterial colonization of mouse organs after i.n. infection. Dissemination of *Y. pestis* strains into the blood and to the internal organs following i.n. inoculation with 1×10^5^ cfu of either Kimberley53 (closed symbols) or Kim53Δ*nlpD* (closed symbols). Mice were sacrificed at the indicated time points, and the bacterial load in the indicated organs was determined as described above.

In an attempt to evaluate the level of attenuation of Kim53Δ*nlpD*, we used mice to monitor the colonization of internal organs by Kim53Δ*nlpD* and the wild type strain Kimberley53. A dramatic disparity between the two strains was observed following infection via both the s.c. and the airway routes. In contrast to the wild type strain, which reached high bacterial loads in all organs within 48 hours, Kim53Δ*nlpD* could not be detected in the draining inguinal lymph nodes (I-LN), the spleen, the lungs or the blood of the subcutaneously infected mice at 24 hours post infection (data not shown) and up to 10 days post infection (Fig. 6A). No visible lesions were observed at the site of the injection.

When mice were infected intranasally with Kim53Δ*nlpD*, the bacterial load in the lungs decreased rapidly by two orders of magnitude within 48 hours to an average of 110 cfu (Fig. 6B), and bacteria were cleared within 3 days post infection (data not shown). In contrast, the bacterial load increased rapidly when the wild type strain was used. Moreover, Kim53Δ*nlpD* was unable to colonize the mediastinal lymph node (M-LN), the spleen or the blood, whereas the number of colony forming units in all organs increased rapidly to an average of 10^4^–10^7^ cfu within 48 hours for the wild type strain (Fig. 6B), causing the death of all mice within 4 days.

These observations indicate that NlpD is required for *Y. pestis* propagation and dissemination to target organs, and they corroborate the non-virulent phenotype of Kim53Δ*nlpD* in both pneumonic and bubonic infection models.

### *In vitro* characteristics of the *nlpD*-null mutant

**Figure 7:**
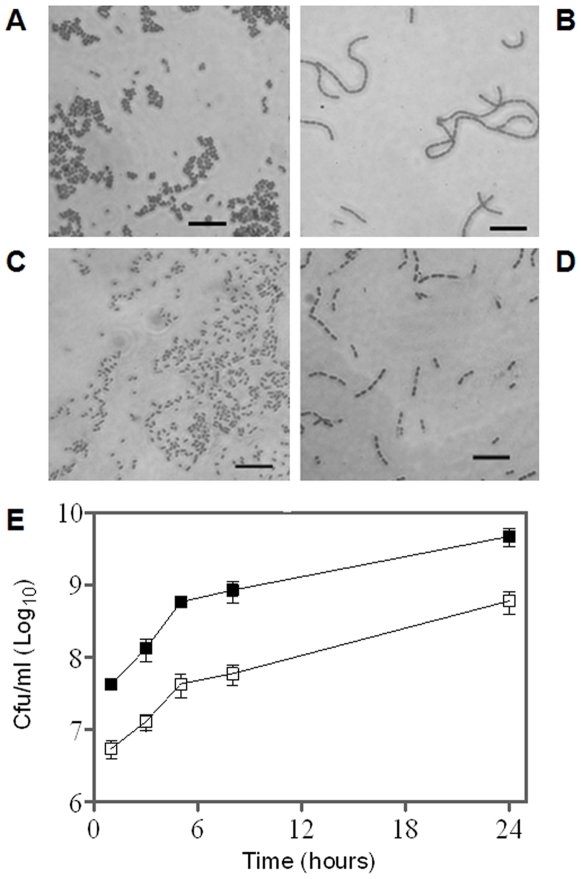
Growth curves and microscope analyses of *Y. pestis* strains following *in vitro* growth. *Y. pestis* strains were grown for 24 hours at 28°C (A and B) or at 37°C (C and D) in HIB. Gram staining of Kimberley53 (A and C) and Kim53Δ*nlpD* (B and D) was performed and bacilli were observed by light microscopy at a magnification of ×1000. Scale bar = 10 µm. (E) Growth curves of Kimberley53 (closed squares) and Kim53Δ*nlpD* (open squares). Bacteria were inoculated (initial OD_660_ = 0.05) and grown at 37°C in HIB for 48 hours. Cultures were sampled at the indicated time points after inoculation and cfu values were determined by plating on BHI agar.

Bacterial lipoproteins are components of the cell envelope of Gram-negative bacteria and are usually localized at the periplasmic space anchored to either the outer or the inner membrane [41]. *Y. pestis* NlpD has a threonine residue immediately after the fatty-acylated cysteine and is therefore predicted to reside in the outer membrane similarly to the *E. coli* NlpD [41], [42]. Microscope analyses indicated that *in vitro* culturing of Kim53Δ*nlpD* at 28°C leads to formation of unsegmented chains containing an average of 7.2±5.6 cells/chain as opposed to the more common aggregative morphology of Kimberley53 (Figures 7B and 7A, respectively). Elevation of the culturing temperature to 37°C (Fig. 7D), reduced significantly the number of Kim53Δ*nlpD* cells/chain to 4±2.5 (T-test P<1E-14), while the wild type morphology is temperature independent (Fig. 7C). In spite of this change, NlpD is not essential for *Y. pestis* cell growth, at least under laboratory conditions, as shown by the normal growth rate of the *nlpD* mutant (Fig. 7E). Growth rate of Kim53Δ*nlpD* was found to be similar to that of the wild type strain under *in vitro* culture conditions at 37°C (Fig. 7E) and at 28°C (data not shown) as determined by ANOVA test of regression (*P*>0.15). Of note is that there is a significant difference between the intercept of the *nlpD* mutant and the wild type strain (*P*<0.05) due to the filamentous phenotype of the mutant (Fig. 7B and 7D). Complementation of *nlpD* expression eliminated completely the abnormal cell morphology and restored the wild type morphology (data not shown).

**Figure 8:**
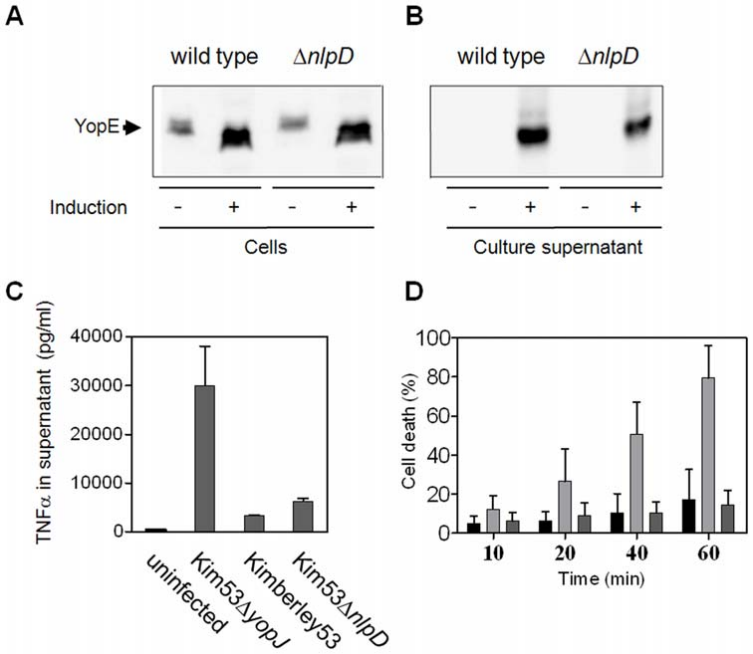
Analysis of TTSS activity and resistance to acidic pH of *Y. pestis* strains. To assay the functionality of the TTSS, wild type Kimberley53 and avirulent Kim53Δ*nlpD* strains were grown for 4 hours at inducing and non–inducing conditions (37°C±calcium). Western blot analyses of bacterial extracts (A) and culture supernatants (B) were performed with anti-YopE antibodies. (C) Suppression of TNF-α secretion from infected macrophages. RAW264.7 cells were infected by impaction with 100 MOI of Kimberley53, 50 MOI of Kim53Δ*nlpD* or 100 MOI of Kim53Δ*yopJ*. Secretion of TNF-α into the culture medium was monitored 2 h after initiation of infection by ELISA. (D) Resistance to acidic pH. Overnight cultures of Kimberley53 (black columns), Kim53Δ*nlpD* (dark gray columns) and Kim53Δ*nlpD*(p*nlpD*) (light gray columns) were used to inoculate HIB (initial OD_660_ = 0.1). These cultures were incubated at 37°C for 4 hours, washed with phosphate buffered saline and then exposed to acidic stress (pH 4.2) for the indicated time. Viable cell counts were determined by plating dilutions on BHI agar and incubating at 28°C for 48 hours.

It can be assumed that deletion of *nlpD* in *Y. pestis* may influence membrane-related functions, such as TTSS activity, which is known to be essential for *Y. pestis* virulence, and thus could lead to the attenuated phenotype. However, preservation of TTSS functionality, at least *in vitro*, was demonstrated by retention of calcium-dependent growth at 37°C and expression and secretion of Yop effectors such as YopE (Fig. 8A and 8B) and YopJ (data not shown), into the culture medium following exposure to inducing conditions. Moreover, the *nlpD*-null mutant preserves the ability to translocate Yop effectors into host cells, like the wild type strain, as evidenced by suppression of TNF-α secretion from infected RAW264.7 macrophages (Fig. 8C). The genes flanking *nlpD* were found in many enteropathogens to be involved in survival during stationary phase and under other environmental stress conditions [36]. In search of a possible explanation for the attenuation of Kim53Δ*nlpD*, we evaluated the involvement of *Y. pestis* NlpD in resistance to prolonged growth in rich (HIB) and minimal (M9) broth, and in acidic and oxidative conditions that simulate the intra-phagosomal milieu. Exposure of Kim53Δ*nlpD* and Kimberley53 to prolonged *in vitro* growth (up to 96 h), and to oxidative stress (10–50 mM H_2_O_2_) indicated that NlpD is not essential for resistance to these conditions (data not shown). In contrast, Kim53Δ*nlpD* was slightly more susceptible than wild type Kimberley53 to acidic pH (Fig. 8D). Moreover, the Kim53Δ*nlpD*(p*nlpD*) strain, in which NlpD expression was restored, regained resistance to these conditions (Fig. 8D). The possible linkage between acidic susceptibility and attenuation of Kim53Δ*nlpD* awaits further study.

### Evaluation of the immunization potential of the Kim53Δ*nlpD* strain

The high level of attenuation of Kim53Δ*nlpD* motivated us to evaluate the potential of this defined mutant to serve as a vaccine. In a model of bubonic plague, mice were injected subcutaneously with 1×10^5^ cfu of either Kim53Δ*nlpD* or the *Y. pestis* EV76 prototype vaccine strain. Fifty days later, mice were challenged subcutaneously with 1×10^5^ LD_50_ of the fully virulent Kimberley53 strain. As shown in Table 3, the geometric mean ELISA titers of αF1 and αV antibodies measured in sera from the mice at 30 days post immunization with Kim53Δ*nlpD* were 17,100 and 26, respectively. Similar αF1 and αV titers were measured following immunization with Kim53-K9. In contrast, mice vaccinated with the EV76 strain demonstrated anti-F1 geometric mean titers of less than 30 and anti-V antibody ELISA titers below the limit of detection (Table 3). These results suggest that although Kim53Δ*nlpD* was highly impaired in colonization of host internal organs, its persistence within the host lasted long enough for the host to mount an effective immune response. Consistent with these results, all mice vaccinated with Kim53Δ*nlpD* survived s.c. challenge with 1×10^5^ LD_50_ of Kimberley53 without showing signs of illness whereas a similar dose of EV76 failed to elicit significant protective immunity (Table 3). The EV76 strain appears to be effective only when applied at a high vaccination dose (10^7^ cfu, Flashner *et al* 2004).

**Table 3:** Vaccine potential of the *Y. pestis* Kim53Δ*nlpD* strain against bubonic plague.

Immunization^a^	Antibody response^b^ GMT (GeoStDv)				Percent survival^c^ (alive/total)	
	αF1		αV			
Kim53-K9	9,250	(2.0)	93	(7.7)	90	(9/10)
Kim53Δ*nlpD*	17,100	(3.4)	26	(29.3)	100	(11/11)
EV76	29	(4.2)	<10	-	10	(1/10)
Control	<10	-	<10	-	0	(0/10)

a Mice were immunized subcutaneously with 10^5^ cfu of the indicated *Y. pestis* strains.

b Anti-F1 and anti-V titers were determined by ELISA.

c Fifty days post immunization, mice were challenged subcutaneously with 10^5^ LD_50_ of the virulent *Y. pestis* Kimberley53 strain (1 LD_50_ = 1–3 cfu).

In the mouse model of pneumonic plague, a single s.c. immunization with 1×10^5^–1×10^7^ cfu of Kim53Δ*nlpD* or EV76 was followed 50 days later by i.n. challenge of virulent *Y. pestis* Kimberley53 (Table 4). The challenge dose was 5,500 cfu, which is equivalent to 10 LD_50_. While all control mice died within 4 days, a protection level of 33% was obtained following immunization with 10^5^ cfu of Kim53Δ*nlpD*. Higher protection levels of 66% and 82% were obtained by immunization with increasing doses of Kim53Δ*nlpD* (10^6^ cfu and 10^7^ cfu, respectively). In contrast, EV76 was unable to elicit protection following immunization with 10^5^ cfu, and a protection level of 33% was attained only after the immunization dose was increased to 10^7^ cfu (Table 4). Comparing total survival in the *nlpD*-vaccinated mice to EV76-vaccinated mice by Chi-square analysis revealed highly significant difference (P = 0.0013). These findings strongly accentuate the potency of Kim53Δ*nlpD* in establishing effective protective immunity and suggest that it may be a suitable platform for a live vaccine.

**Table 4:** Vaccine potential of the *Y. pestis* Kim53Δ*nlpD* strain against pneumonic plague.

Immunization^a^		Antibody response^b^ GMT (GeoStDv)				Percent survival (alive/total)^c^	
*Y. pestis* strain	Dose (cfu) d	αF1		αV			
	10^5^	4,500	(10.5)	10	(3.6)	33	(2/6)
Kim53Δ*nlpD*	10^6^	31,800	(4.8)	32	(36)	66	(4/6)
	10^7^	7,200	(4.6)	12	(28)	82	(9/11)
	10^5^	20	(4.9)	<10	-	0	(0/6)
EV76	10^6^	100	(15.3)	<10	-	17	(1/6)
	10^7^	635	(4.2)	18	(21)	33	(2/6)
Control	-	<10	-	<10	-	0	(0/6)

a Mice were immunized subcutaneously with the indicated dose of each strain.

b Anti-F1 and anti-V titers were determined by ELISA.

c Fifty days post immunization, mice were challenged intranasally with 10 LD_50_ of the virulent *Y. pestis* Kimberley53 strain (1 LD_50_ = 550 cfu).

d Under the specific experimental conditions, one colony forming unit of Kim53Δ*nlpD* is estimated to be 1-6 bacilli.

## Discussion

Studies performed in the last decade with several Gram negative bacteria have demonstrated that the genomic region including the *surE*, *pcm, nlpD* and *rpoS* genes is important for survival under environmental stress conditions [18], [36], [43], [44]. In the present study, we have further characterized the *Y. pestis pcm* locus genes and analyzed their expression and involvement in the pathogenesis of *Y. pestis* using mouse models of bubonic and pneumonic plague. Systematic deletion mutagenesis of the *surE, pcm, nlpD* and *rpoS* genes and complementation studies allowed us to identify the NlpD lipoprotein as the only essential factor for *Y. pestis* pathogenesis in this locus.

Subcutaneous and intranasal administration of a *Y. pestis nlpD*-null strain to mice demonstrated that this strain is severely attenuated (LD_50_>10^7^cfu, Table 2), and is impaired in its ability to colonize internal organs (Fig. 6). In addition, the *nlpD*-null mutant was unable to produce a systemic disease following intravenous inoculation with 10^6^ cfu and infected mice lacked any visible disease symptoms (data not shown).

Trans-complementation experiments verified that NlpD is an essential *Y. pestis* virulence factor (Fig. 5C, Table 2). Using a similar strategy, virulence was also restored to the original transposon insertion mutant (Kim53-K9), confirming that its attenuation resulted from elimination of *nlpD* expression and not from disruption of *pcm* expression (Fig. 5C, Table 2; note also that the Kim53Δ*pcm* is as virulent as the wild type strain). To the best of our knowledge, this is the first demonstration of a single chromosomal *Y. pestis* factor that is essential for development of both bubonic and pneumonic plague.

*Yersinia enterocolitica* NlpD was also suggested to be involved in pathogenesis [45]. In the latter study, transposon insertion within the *Y. enterocolitica nlpD* gene led to attenuation of virulence in a mouse infection model. Interestingly, as opposed to the dramatic attenuation of the *Y. pestis nlpD* mutant shown in the present study, the *Y. enterocolitica nlpD* mutant displayed only a limited impairment of virulence [45]. This finding might imply that *Y. pestis nlpD* is required for an activity specific to the development of plague disease.

Recently, an additional *Y. pestis* lipoprotein, Braun's lipoprotein (Lpp), was found to be important for virulence [40]. It should be noted, however, that in contrast to NlpD, Lpp contributes only to the production of bubonic plague but not to that of the pneumonic plague [40]. Lipoproteins have been implicated in the pathogenesis of other bacterial pathogens as well. For example, disruption of the *lsp* and *lgt* genes, which are involved in lipoproteins metabolism, caused attenuation of virulence in *M. tuberculosis*, *S. aureus* and *S. pneumoniae* [46]–[48].

The dramatic loss of virulence of the *Y. pestis nlpD*-null strain is reminiscent of the non-virulent phenotype of *Y. pestis* strains lacking the pCD1 plasmid that carries the genes encoding the type III secretion system. However, our results indicate that the *nlpD* mutant is able to properly express and secrete Yop effectors during *in vitro* inducing conditions (Fig. 8A and 8B) and is able to translocate effectors into host cells, as reflected by its ability to suppress TNFα secretion from infected macrophages (Fig. 8C). Moreover, the V-antigen multifunctional virulence factor important for type III secretion system activity is expressed by the *nlpD* mutant during infection as reflected by development of αV antibodies following immunization of mice (Table 3 and 4). Taken together, these observations suggest that the type III secretion system is functional in the *nlpD* mutant.

The finding that the *Y. pestis nlpD* gene is expressed from control elements shared with either *pcm* or *rpoS* raised the possibility that NlpD might also be important for survival under environmental stress conditions. However, the *nlpD*-null mutant did not differ significantly from the wild type strain in the ability to survive during prolonged growth in rich and minimal broth and during exposure to oxidative conditions. Yet, we found a slight increase in the mutant's sensitivity to *in vitro* acidic conditions (Fig. 8), similar to the conditions present in the intra-phagosomal milieu. While the latter does not seem to account for the dramatic loss of virulence, further studies are needed to understand the importance of the sensitivity of the mutant to acidic conditions.

**Figure 9:**
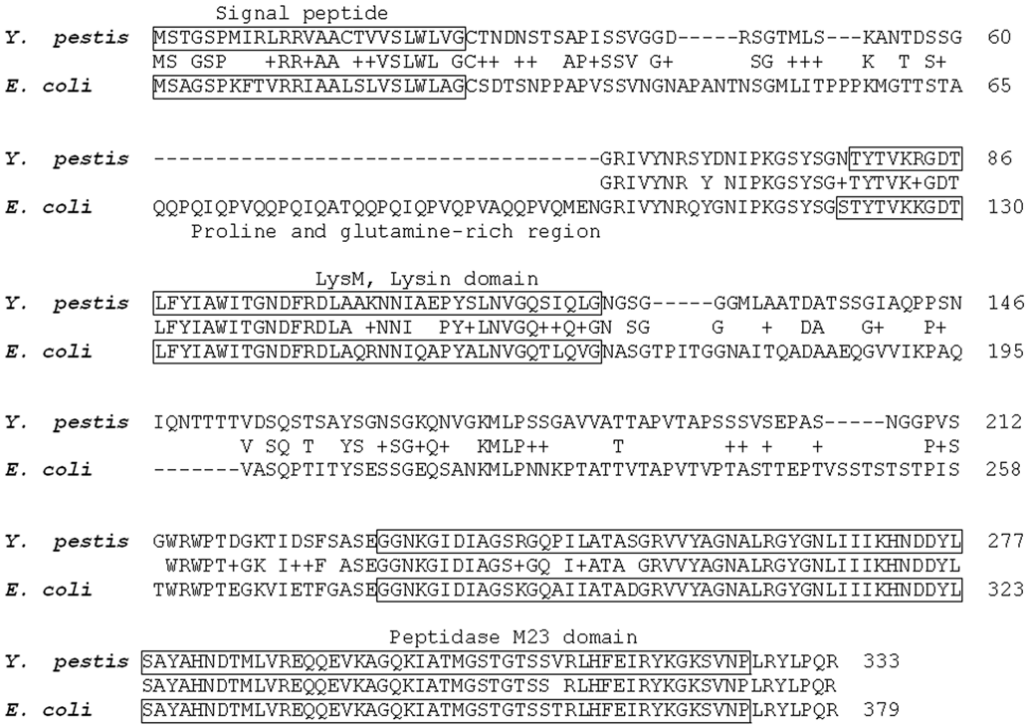
Alignment of amino acid sequences of the *Y. pestis* and *E. coli* NlpDs. Amino acid sequence identities/homologies between the NlpDs of *Y. pestis* Kimberley53 and *E. coli* K12. The NlpD signal peptide region and the LysM and M23 domains are boxed; the P/Q rich region is underlined.

The aberrant shape of the *Y. pestis* Δ*nlpD* bacilli (Fig. 7B and 7D) suggests that NlpD is important for cell separation. Consistent with this assumption is the structure of NlpD (Fig. 9), which contains an N-terminal LysM domain found in a variety of enzymes involved in bacterial cell wall degradation [49]–[51]. NlpD also contains a C-terminal M23 metallopeptidase region (Fig. 9). The M23 family of endopeptidases includes proteins that are involved in bacterial cell separation [52], however, no proteolytic activity has been demonstrated for bacterial lipoproteins of this family (see the MEROPS peptidase database, http://merops.sanger.ac.uk [53]). Of note is that *in vitro* cell growth of the *nlpD* mutant was not affected (Fig. 7E). The observed phenotype of the *nlpD-*null mutant (Fig. 7B and 7D) suggests a linkage between impairment of cell separation and attenuation of virulence. Interestingly, similar links have already been described for other bacterial pathogens [54], [55]. Furthermore Kajimura and colleagues [54] hypothesized that cluster formation of the attenuated *S. aureus* Sle1 mutant, which lacks the lysM-containing *N*-acetylmuramyl-L-alanine amidase, inhibits the dissemination of daughter cells and thus could affect the spread of the bacteria during infection. Indeed, the *Y. pestis nlpD* mutant is totally impaired in its ability to disseminate from the site of infection to the internal organs (Fig. 6).

Interestingly, the highly attenuated phenotype of the *nlpD* mutant and its inability to colonize host organs did not seem to prevent the development of immunity against plague following s.c. infection. Rather, this strain seemed to effectively stimulate a long-term adaptive immune response as demonstrated by the generation of high F1 antibody titers. Accordingly, immunization of mice with low doses of Kim53Δ*nlpD* resulted in remarkably higher humoral response and protection levels against bubonic and pneumonic plague than did immunization with the *Y. pestis* EV76 vaccine strain (Tables 3 and 4). The differential behavior of the two vaccine strains may result from the complete absence of the chromosomal *pgm* locus (102 kb including the pathogenicity island), from EV76. The contribution of the *pgm* locus to *Y. pestis* survival in host cells is well documented [56]. The observed development of protective immunity could have practical implications in the design of future *Y. pestis* vaccines or therapies against both bubonic and pneumonic plague.

## Materials and Methods

### Bacterial strains, plasmids, mutant construction and routine growth conditions

The *Y. pestis* strains and the plasmids used in this study are listed in Table 1. The primers used for construction of the *Y. pestis* derivatives are listed in Table S1. Construction of the Kimberley53 deletion mutants was performed by replacing part of the gene coding sequence with a linear fragment containing the Kan^r^ GeneBlock™ resistance cassette (pUC4K plasmid, Pharmacia) by homologous recombination, using previously established methodologies [60]. In all constructs the Kan^r^ resistance cassette is oriented in the same direction as the *pcm* locus genes (Fig. 2A). To preserve expression of downstream genes, the resistance cassette does not include transcription terminator sequences.

Genotype verification of all obtained phenotypes was done by PCR and Western blot analysis. All of the Kimberley53-derived strains carry the pMT1, pCD1, pPCP1 plasmids and the *pgm* locus.

Bacteria were isolated from stocks on selective BIN medium [61]. Routine propagation was performed on brain heart infusion agar (BHIA) (Difco) or HIB (Difco). For generation of *nlpD*-complemented strains, a 1444 kb fragment extending from the 3′ end of the *nlpD* gene up to 442 nt upstream of the ATG start codon of *nlpD* was amplified from the DNA of the virulent *Y. pestis* Kimberley53 strain. The resulting PCR product was cloned into pBR322 between the NheI and PstI sites and its sequence was verified. Plasmids were introduced into the Kim53Δ*nlpD* and Kim53-K9 strains by electroporation to give the Kim53Δ*nlpD*(p*nlpD*) and Kim53-K9(p*nlpD*) strains. These strains were routinely grown in media supplemented with 100 µg/ml ampicillin (Sigma, Israel).

### DNA sequence determination of the *Y. pestis* Kimberley53 *pcm* locus and *in-silico* analysis

The *Y. pestis* CO92 sequence corresponding to the *pcm* locus (nt 3744222 to 3747841) was compared to the Kimberley53 sequence at the equivalent region and was found to be identical) GenBank acc. no. FJ666123). All sequencing reactions were performed with an ABI310 genetic analyzer (Applied Biosystems) using the ABI PRISM BigDye terminator reaction kit. The genome alignment of the orthologous regions comprising the *surE, pcm, nlpD* and *rpoS* genes was generated by the multiple genome alignment software Mauve [62]. The sequences were extracted from the following GenBank (NCBI) entries: NC_003143 (*Y. pestis* CO92), NC_003197 (*S. typhimurium* LT2), NC_000913 (*E. coli* K12).

### Growth curves

*Y. pestis* strains were grown by shaking (100 rpm) at 28°C in HIB supplemented with 0.2% (+)xylose (Sigma) and 2.5 mM CaCl_2_ (Sigma). The resulting cultures were diluted in fresh broth to an OD_660_ of 0.01 and allowed to grow for 48 hours at either 28°C or 37°C while shaking at 180 rpm. At the indicated time points, aliquots were removed for determination of the OD_660_ and the number of colony forming units (cfu) after plating on BHI agar plates and incubation at 28°C for 48 hours. Experiments were repeated for three times. The growth of *Y. pestis* strains under nutrient-limiting conditions was assessed in M9 medium at pH 8.0 [0.05% NaCl (Merck), 0.1% NH_4_Cl (Merck), 0.3% KH_2_PO_4_ (Merck), 0.7% Na_2_HPO_4_ (Merck) 10^−4^ M CaCl_2_ (Merck), 10^−3^ M MgSO4 (Merck), 0.4% D-(+)-glucose (Sigma), 5 µg/ml thiamine (Sigma), 50 µg/ml L-arginine (Sigma), 50 µg/ml L-cysteine (Sigma), 50 µg/ml glycine (Sigma), 50 µg/ml L-methionine (Sigma), 50 µg/ml L-phenylalanine (Sigma)]. Aliquots of the liquid cultures were taken at different time points for cfu enumeration and Western blot analysis. Comparison of the *Y. pestit* strain's growth rates was performed by log-log transformation. The slop of the curves was compared using an ANOVA test of regression.

### Western blot analysis

Bacteria (OD_660_ = 0.1) were lysed with Laemmli Sample buffer (Bio-Rad) and protein concentrations were determined using bicinchoninic acid (BCA Protein Assay Reagent, Pierce) according to the manufacturer's instructions. Equal amounts of protein were loaded and separated by 12% SDS-PAGE. After transfer to nitrocellulose membranes, duplicate membranes were developed with rabbit polyclonal anti-peptide Pcm and NlpD antibodies, with rabbit polyclonal *S. typhimurium* RpoS antibodies (a generous gift from François Norel, Institute Pasteur), or with goat anti-YopE antibodies (bL-20, Santa Cruz Biotechnology) and with rabbit polyclonal anti-peptide YopJ [60]. Probing with the primary antibody was followed by incubation of the membranes with HRP-conjugated second antibody, and then the reactive protein bands were visualized by ECL. The Pcm and NlpD anti-peptide antibodies were raised by immunizing rabbits with maleimide-activated KLH (Pierce) conjugated to the synthetic peptides CERLLQAIEAVPRER (amino acids 21–34 of Pcm) and CVGGDRSGTMLSKANT (amino acids 39–53 of NlpD). Antibodies were affinity purified using a Sulfolink-peptide coupling gel column (Pierce) according to manufacturer's instructions.

### Total RNA isolation, primer extension and RT-PCR analyses

HIB supplemented with 0.2% (+)xylose and 2.5 mM CaCl_2_ was inoculated with *Y. pestis* bacteria (initial OD_660_ = 0.01), and incubated for 24 hours at 28°C or 37°C with shaking at 100 rpm. Total RNA was isolated using a RiboPure^TM^-bacteria Kit (Ambion) and was then treated with DNase I (Ambion) according to the manufacturer's instructions.

Primer extension (PE) reactions were carried out according to Lloyd and colleagues [63]. Briefly, for each reaction, FAM-labeled primer (Table S1, final concentration 5–10 nM) was added to 20 µg of total RNA, and first strand cDNA synthesis was performed using the AMV RT enzyme (Promega). FAM-labeled cDNAs were cleaned using a Preforma® DTR Gel Filtration Cartridge (Edge BioSystems) according to the manufacturer's instructions and then air-dried with a heated SpeedVac centrifuge and resuspended in 25 µl of UltraPure™ Formamide (Invitrogen) with 1.5 µl MapMarker®1000 (BioVentures, Inc.). The mixture was heated to 90°C for 2 minutes, chilled on ice for 5 minutes and then used for electrophoresis, using an ABI310 genetic analyzer (Applied Biosystems). The DNA fragments were sized using the GeneScanR Analysis Software version 3.1 (Applied Biosystems). For reverse transcriptase-PCR analyses (RT-PCR), primers were added to 1 µg of total RNA and synthesis was carried out with AMV RT (Promega) at 42°C for 1 hour according to the manufacturer's instructions. After first strand synthesis the cDNA was diluted 1:2 and an aliquot (3 µl) was taken for PCR amplification. Positive and negative control reactions were performed with 200 ng of DNA or with total RNA, respectively.

### Infection of mice

All experiments were performed in accordance with the Israeli law and were approved by the Ethics Committee for animal experiments at the Israel Institute for Biological Research. Female OF1 mice, 5–6 weeks old (IFFA CREDO S.A., France), were used in all infection studies. For s.c. infections of mice, the *Y. pestis* strains were grown on BHIA for 48 hours at 28°C. Bacterial colonies were harvested from the BHIA plates at the end of the incubation period, suspended in 5 ml of saline solution (0.9% NaCl) and diluted in saline solution to the required infection dose. Bacteria were quantified by counting colony forming units after plating and incubating on BHI agar plates. In s.c. infections, samples of 100 µl were administered into the lower back of the mice. Under these conditions, the LD_50_ of the Kimberley53 strain is 1–3 cfu [4]. For i.n. infections, the *Y. pestis* strains were grown on BHIA for 48 hours at 28°C. Bacterial colonies were suspended in HIB supplemented with 0.2% (+)xylose and 2.5 mM CaCl_2_ to give an initial OD_660_ of 0.01, and the cultures were incubated for additional 22 hours at 28°C or 37°C with shaking at 100 rpm. At the end of the incubation period, the cultures were washed, diluted in saline solution to the required infection dose and quantified by cfu counting as described above. Prior to infection, mice were anaesthetized with a mixture of 0.5% ketamine HCl and 0.1% xylazine injected intraperitoneally and were then infected intranasally with 35 µl of the bacterial suspension. The LD_50_ experiments were performed with groups of 8 mice (i.n.) or 5 mice (s.c.). The LD_50_ values were calculated according to the method described by Reed and Muench [64], protection analyses were performed by Chi-square test.

### Monitoring disease progression

Mortality rates were expressed in accordance with the required information by either the proportion of dead animals at the end of the monitoring period (14 days unless otherwise indicated), by MTTD, or by the 50% lethal dose (LD_50_). Monitoring of disease progression was performed by tracking bacterial dissemination to the internal organs and blood. In s.c. infection experiments, organs were aseptically removed 48 hours after infection with 1×10^4^ cfu of the wild type Kimberley53 strain (a terminal stage of disease) or with 1×10^5^ of Kim53Δ*nlpD*. Groups of four mice were anaesthetized, tail vein blood was collected and spleens, lungs and draining inguinal lymph nodes (I-LN) were harvested. Tissue homogenates were prepared in 1 ml PBS/organ. Bacterial enumeration in tissue homogenates or in blood samples was done by plating serial dilutions in PBS on BHI agar and calculating the cfu/organ or cfu/1 ml blood. In i.n. infection experiments, groups of four mice were anesthetized, and tail vein blood was collected one hour and 48 hours post infection with 1×10^5^ cfu. The lungs, spleens and the mediastinal lymph nodes (M-LN) were harvested and prepared as described above for bacterial enumeration.

### Morphology analysis

*Y. pestis* bacilli were observed by light microscopy using a Nikon Eclipse E200 microscope at a magnification of ×1000. Gram staining was performed using a HT90A kit (Sigma-Aldrich) according to the manufacturer's instructions. *Y. pestis* cells in at least 20 random non-overlapping microscopic fields were counted.

### TTSS functionality analyses and stress survival assays

For secretion of TTSS proteins, bacteria were grown at 28°C in HIB for 18 hours with shaking at 150 rpm. The bacteria were then diluted to an OD_660_ of 0.05 in HIB medium supplemented with either 20 mM sodium oxalate (Sigma) and 20 mM MgCl_2_ (Sigma) (inducing conditions) or 2.5 mM CaCl_2_ (non-inducing conditions) and were grown for additional 4.5 hours at 37°C with shaking at 100 rpm. Cultures were centrifuged at 4500 g for 10 min, the supernatants were collected and the proteins were precipitated with 10% TCA overnight at 4°C and the subjected to SDS-PAGE. Analysis of the suppression of TNF-α secretion from macrophages was preformed as described in Zauberman *et al.* [60]. Briefly, RAW264.7 macrophages (2×10^5^) were seeded in 24-well plates and infected at the indicated MOI (determined by the cfu count) for 2 hours. The concentration of TNF-α in the culture medium was determined by ELISA using the DuoSet mouse TNF-α immunoassay system (R&D systems). The susceptibility of the *Y. pestis* strains to acidic pH was determined by plating stationary-phase bacteria on BHI agar after incubation for 10–60 minutes in 1×PBS adjusted to pH 4.2 with citric acid. The susceptibility of the *Y. pestis* strains to hydrogen peroxide was determined by plating stationary-phase bacteria on BHI agar after exposure to hydrogen peroxide (10–50 mM in 1×PBS, pH = 7) for up to 60 minutes.

### Antisera and serological tests

Mouse anti-F1 and anti-V antigen IgG antibody titer determinations were performed by ELISA as previously described by Flashner and colleagues. [4]. Briefly, microtiter plates were coated with purified recombinant F1 antigen [65] or with 500 ng of purified Glutathione-S-Transferase -V- antigen fusion protein prepared according to Leary *et al*. [66]. The tested sera were serially diluted by 2-fold dilutions in a final volume of 50 µl and were incubated in the wells for 1 hour at 37°C. Alkaline phosphatase–labeled rabbit anti-mouse IgG (1/2500 dilution, Sigma) was used as the secondary antibody. Titers were defined as the reciprocal values of the endpoint serum dilutions, which had OD_405_ values two fold higher than the normal serum controls.

## Supporting Information

Figure S1 Putative *Y. pestis rpoS* promoter sequences identified *in silico*. (A) Sequences of *rpoS* promoters in different bacteria; the consensus −35 sequences is indicated by ambiguity code. (B) The DNA region upstream of the Kimberley53 and CO92 *rpoS* coding sequences, containing the putative −10 and/or −35 promoter sequences.
(0.73 MB TIF)

Figure S2 Organization and expression of *Y. pestis pcm* locus genes in wild-type and mutant strains. Transcription start sites (TS) within *Y. pestis pcm* locus are depicted. Deleted region are represented by dashed line and replaced by a kanamycin resistance cassette. The expression level of each gene is indicated by the intensity of its color: dark gray - comparable to the wild type strain, light gray - lower than the wild-type strain, colorless - no expression. The mini-Tn5 transposon inserted within Kim53-K9 is indicated by a black arrow (the arrow points to the direction of transcription of the kanamycin resistance cassette). The (*) symbol indicates a putative transcription start site (see also in Results).
(0.60 MB TIF)

Table S1 Sequences of primers used in this study. a. Numbers in parenthesis indicate the position of the primer relative to the first nucleotide in the ORF. b. Underlined bases indicate restriction sites for cloning of the amplified sequence. c. FAM (6-carboxyfluorescein) labeled primers for primer extension. These primers where also used for first strand synthesis in the RT-PCRs.
(0.05 MB DOC)
