# Supplementary figures and images for: The NlpD Lipoprotein Is a Novel Yersinia pestis Virulence Factor Essential for the Development of Plague
Source: PLoS One. 2009 Sep 14;4(9):e7023. doi: 10.1371/journal.pone.0007023 (PMC2736372; doi:10.1371/journal.pone.0007023)

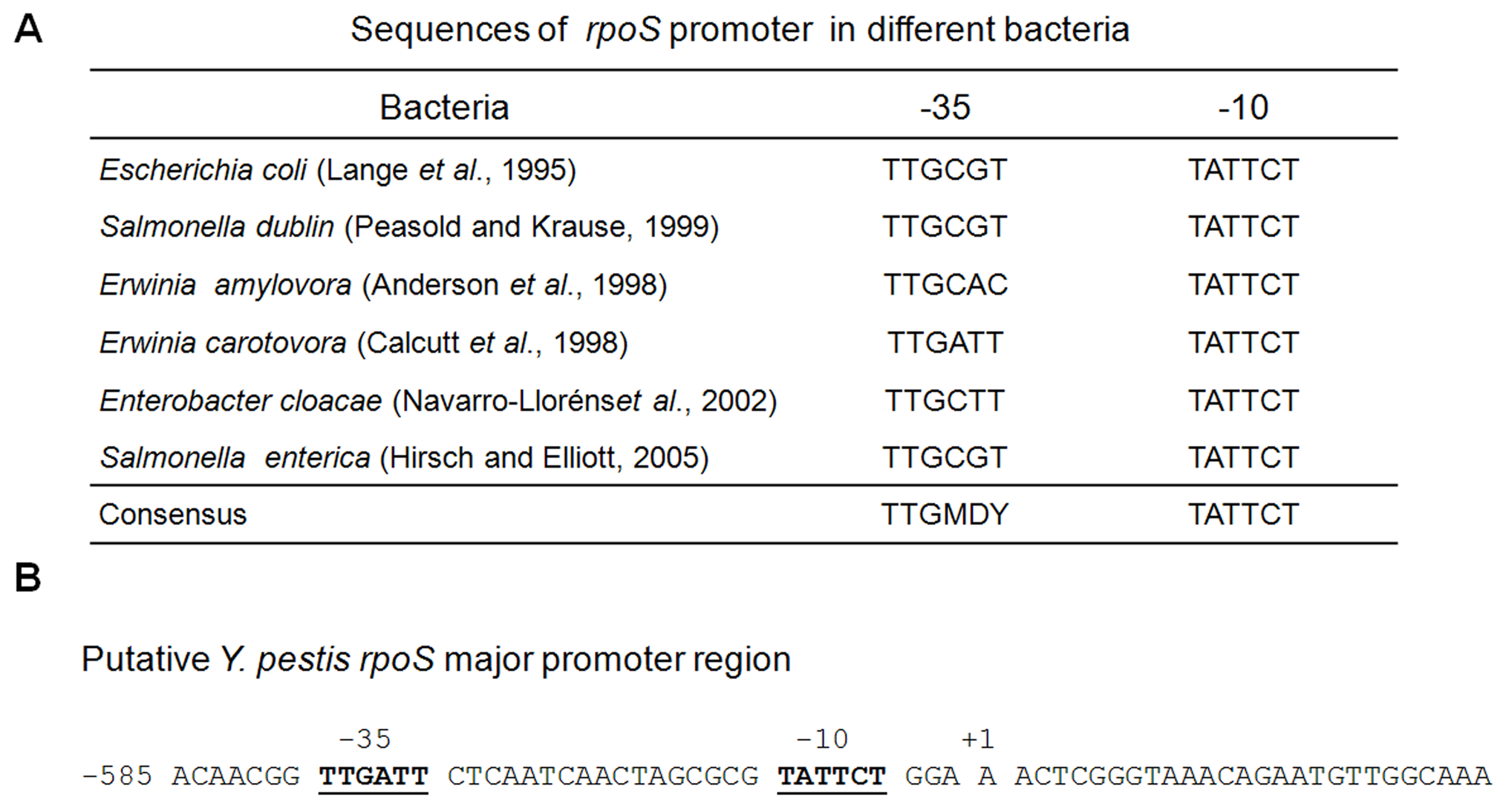

Supplement: Figure S1 — Putative Y. pestis rpoS promoter sequences identified in silico. (A) Sequences of rpoS promoters in different bacteria; the consensus −35 sequences is indicated by ambiguity code. (B) The DNA region upstream of the Kimberley53 and CO92 rpoS coding sequences, containing the putative −10 and/or −35 promoter sequences. (0.73 MB TIF) [file pone.0007023.s001.tif]

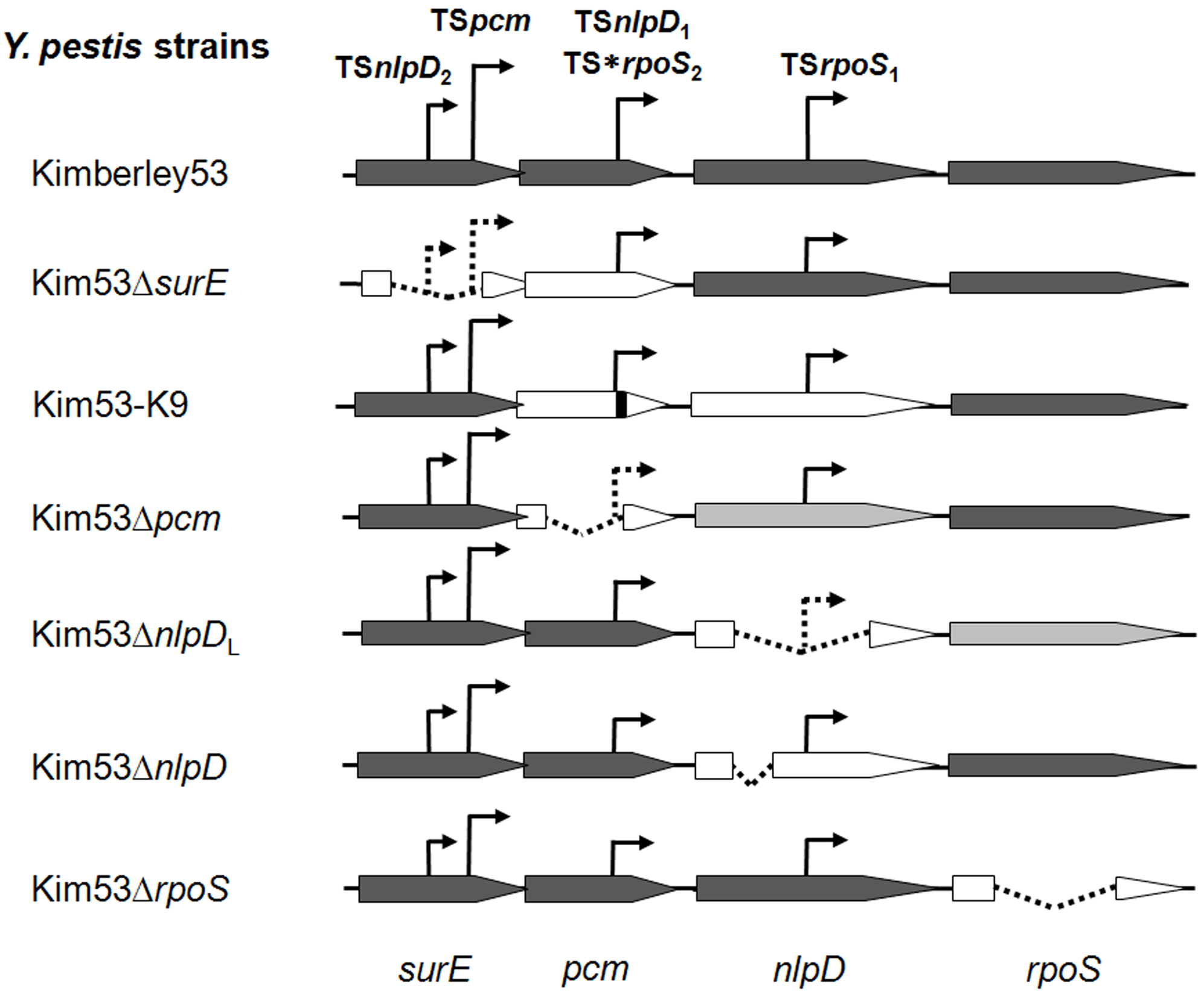

Supplement: Figure S2 — Organization and expression of Y. pestis pcm locus genes in wild-type and mutant strains. Transcription start sites (TS) within Y. pestis pcm locus are depicted. Deleted region are represented by dashed line and replaced by a kanamycin resistance cassette. The expression level of each gene is indicated by the intensity of its color: dark gray - comparable to the wild type strain, light gray - lower than the wild-type strain, colorless - no expression. The mini-Tn5 transposon inserted within Kim53-K9 is indicated by a black arrow (the arrow points to the direction of transcription of the kanamycin resistance cassette). The (*) symbol indicates a putative transcription start site (see also in Results). (0.60 MB TIF) [file pone.0007023.s002.tif]
