# Supplementary material for: The NlpD Lipoprotein Is a Novel Yersinia pestis Virulence Factor Essential for the Development of Plague
Source: PLoS One. 2009 Sep 14;4(9):e7023. doi: 10.1371/journal.pone.0007023 (PMC2736372; doi:10.1371/journal.pone.0007023)
Supplement: Table S1 — Sequences of primers used in this study. a. Numbers in parenthesis indicate the position of the primer relative to the first nucleotide in the ORF. b. Underlined bases indicate restriction sites for cloning of the amplified sequence. c. FAM (6-carboxyfluorescein) labeled primers for primer extension. These primers where also used for first strand synthesis in the RT-PCRs. (0.05 MB DOC) [file pone.0007023.s003.doc]

Table S1. Sequences of primers used in this study

| Primer position | Primer name *^a^* | Sequence | Purpose *^a^* |
| --- | --- | --- | --- |
| 5’ | Δ*surE*-for(148) | 5'GGCACCCGATCGTAACCGTAGTGGCGCTTCCAATGCATTGACTCTGGATCCCGGGTGACTAACTAGGAGGAATAAATCA 3' | Generation of Kim53Δ*surE* |
| 3’ | Δ*surE*-rev(618) | 5'CATAACCTTGTTCAACCGCCGCAAAGTCAGTATCTGGCCCTGCATCATACTGGGGAAATGGACAATAACCCTACG 3' | Generation of Kim53Δ*surE* |
| 5’ | Δ*pcm*-for(125) | 5'CGGTACCGCGTGAGCGTTTTGTCGATGAAGCGTTGGCTCCCGGGTGACTAACTAGGAGGAATAAATGAGCCATATTCAACGGGAAA 3' | Generation of Kim53Δ*pcm* |
| 3’ | Δ*pcm*-rev(510) | 5'GGCGTTGCACGTACTTTAATGTTTGGAACTGCTCACCAACGGGCATTATTCCCTCCAGGTATTAGAAAAACTCATCGAGCATCAAA 3' | Generation of Kim53Δ*pcm* |
| 5’ | Δ*nlpD*-for(110) | 5'CTAGGTGAAGATCCTTTTTGATTTATTCCTCCTAGTTAGTCACCCGGGGCTGATGGGTGCAGATGTTGAATTATCATTGGT 3' | Generation of Kim53Δ*nlpD* and Kim53Δ*nlpD* _L_ |
| 3’ | Δ*nlpD*-rev(318) | 5'CAATTGAATAGATTGCCCCACATTCAGGCTATAGGGTTCTGCAATGGGGAAATGGACAATAACCCTACGAC 3' | Generation of Kim53Δ*nlpD* |
| 3’ | Δ*nlpD*-rev(866) | 5'TTTTGACCCGCCTTCACTTCTTGTTGTTCCATTATTCCCTCCAGGTACTAGGGGAAATGGACAATAACCCTACGAC 3' | Generation of Kim53Δ*nlpD*_L_ |
| 5’ | Δ*rpoS*-for(123) | 5'TAGTAGATGATGAACCTACTGAAAGCGACCCGGGTGACTAACTAGGAGGAATAAATCAAAAAGGATCTTCACCTAGATCC 3' | Generation of Kim53Δ*rpoS* |
| 3’ | Δ*rpoS*-rev(888) | 5'CTGGCGAACACGCTCGCGTGTCAAACCAATCATTATTCCCTCCAGGTACTAGGGGAAATGGACAATAACCCT 3' | Generation of Kim53Δ*rpoS* |
| 5’ | *nlpD*-for(-442) | 5' GGCGTGCTAGCAGCGTCGATCATGTCTGTTCAGTCGAG 3' | Construction of p*nlpD* (*nlpD* gene +5’ 442 bp +NheI site*^b^*) |
| 3’ | *nlpD*-rev(1002) | 5' CTATCTGCAGATAGCTATCGCTGCGGAAGGTAAC 3' | Construction of p*nlpD* (*nlpD* gene +5’ 442 bp + PstI site*^b^*) |
| 3’ | *nlpD*-rev(151) | 5' FAM-CTTTACTCAACATCGTACCTGAACG 3' ^c^ | Primer Extension from nt 151 of *nlpD* ORF and reverse primer for RT-PCR |
| 3’ | *rpoS*-rev(137) | 5' FAM-TCATCGTCTGCTAACTCGCTTTCAG 3' *^c^* | Primer Extension from nt 137 of *rpoS* ORF and reverse primer for RT-PCR |
| 5’ | *nlpD*-for(-972) | 5' CGGGTGACGCGTTGTGGTAGC 3' | RT-PCR from nt (-972) of *nlpD* ATG start codon |
| 5’ | *nlpD*-for(-1389) | 5' GCACCCGATCGTAACCGTAGTG 3' | RT-PCR from nt (-1389) of *nlpD* ATG start codon |
| 5’ | *rpoS*-for(-539) | 5' GTAAACAGAATGTTGGCAAAATGT 3' | RT-PCR from nt (-539) *rpoS* ATG start codon |
| 5’ | *rpoS*-for(-1355) | 5' CATTATTGTAACCGCAGCACCG 3' | RT-PCR from nt (-1355) *rpoS* ATG start codon |
| 5’ | *rpoS*-for(-1493) | 5' TCATGTCTGTTCAGTCGAGCG 3' | RT-PCR from nt (-1493) *rpoS* ATG start codon |
